# Correlates of maternal mortality in developing countries: an ecological study in 82 countries

**DOI:** 10.1186/s40748-017-0059-8

**Published:** 2017-11-07

**Authors:** Tadele Girum, Abebaw Wasie

**Affiliations:** Department of Public health, College of Medicine and Health Sciences, Wolkite University, Wolkite, Ethiopia

**Keywords:** Maternal mortality ratio, Developing countries, Correlates

## Abstract

**Background:**

Maternal mortality is a major public health issue in developing countries due to its shocking magnitude and lower declining pattern. With appropriate strategy and intensive implementation programs, some countries have made remarkable progress, however in developing countries where 99% of maternal death is occurring; little or no progress has been made. Identifying determinants and designing intervention will have important role to overcome the problem. Therefore this study aimed to identify correlates of maternal mortality in developing countries.

**Methods:**

This study was conducted using international data bases of health metrics from 2008 to 2016 using aggregates of health indicator data from WHO, World Bank, UNDP and UNICEF data bases for 82 developing countries. The dependent variable was the maternal mortality ratio, while the independent variable was socio-economic, health care related and morbidity variables. Data was compiled in excel and analyzed using SPSS version 21.

**Results:**

Maternal mortality ratio is very high in developing countries and enormously varies among countries. A significant relationship between the maternal mortality ratio and socio-economic, health care and morbidity indicator variables was observed. There was an inverse and significant correlation of the maternal mortality ratio with Antenatal care coverage, skilled birth attendance, access to an improved water source and sanitation, adult literacy rate, the Gross National Income per capita and positive relation with disease incidence, unmet need and others.

**Conclusions:**

Maternal mortality is correlated with multiples of socio-economic factors, health care system associated factors, disease burden and their complex interactions. Therefore Policy and programs targeted to improve maternal health and reduce maternal deaths should consider population dynamics, socio-economic influence and health system factors that impose a major risk on mothers.

## Background

World Health Organization estimated that 358,000 maternal deaths (800 deaths every day) occurred worldwide in 2008 as a result of pregnancy or childbirth complications, a 34% decline from the levels of 1990. Despite this decline, developing countries continued to account for 99% of the deaths. Sub-Saharan Africa and South Asia alone accounted for 87% of global maternal deaths. The situation is most dire for women in Sub-Saharan Africa, where one in every 160 women dies of pregnancy related causes during her lifetime, compared with only 1 in 3700 women in developed regions [1–3].

Maternal mortality is much higher in developing countries compared to developed nations owing to lack of adequate medical care; high prevalence of infectious diseases,higher total fertility rate and due to health care system difference. Countries with high maternal mortality ratio have less reliable vital statistics registry system; as a result level of maternal mortality is usually underestimated and little information is available regarding locally specific risk factors for maternal death [2, 3].

More than 70% of all maternal deaths are due to hemorrhage, infection, unsafe abortion, hypertensive disorders of pregnancy, and obstructed labor and the underlying causes for these deaths are poverty, inadequate, inaccessible, or unaffordable health care, unequal access to resources, low status of women and illiteracy [2, 4].

Besides the higher effect on health, Pregnancy related deaths and disabilities also result in losses to social and economic development. The women who die are in the prime of life responsible for the health and wellbeing of their families. Their death represents a drain on all development efforts [5]. Above and beyond the social and economic rationale for preventing this burden of mortality and morbidity is moral imperative [6]. Therefore reducing the high maternal mortality in the developing world should be considered a key policy issue for health, social and economic development.

Fortunately, the vast majority of maternal and newborn deaths can be prevented with proven interventions [7]. It is difficult to establish a causal relationship between maternal mortality and its predictors as a result of rare occurrence of maternal death events. Due to the aforementioned factor identifying determinants of maternal death is a difficult job [8, 9].

Many studies assessed determinants of maternal mortality in a fragmented scope with few cases of maternal death without acknowledging the impact of broader health and social indicators like contraceptive prevalence rate, total fertility rate, literacy rate, Gross national per capita income (GNI) and others on maternal mortality. Therefore this study aimed to assess factors associated with maternal mortality in developing countries using most recent health and health related, social, economic and developmental indicators.

## Methods

### Study design, settings and population

This ecological study was conducted using international data bases of health metrics from 2008 to 2016 from WHO, World Bank, UNDP and UNICEF data bases to identify correlates of maternal mortality in developing countries. Each Developing country is considered as unit of analysis. Developing countries are those with gross national income (GNI) per capital in atlas method of <4125 USD in 2014 which are further categorized as low income counties with GNI of 1045 USD or less and low middle income counties with GNI of 1045–4125 USD. There are 31 low income and 51 lower middle income countries a total of 82 developing countries in 2016 [10].

### Sources of data and data collection procedure

International country-level data is collected by specialized agencies of the United Nations (such as WHO for health data), and other intergovernmental organizations such as World Bank Data and UN data which compile data from many different sources.

Country-specific estimates for maternal mortality were based on the most recent interagency estimates. For 62 Member States with relatively complete data from national death registration systems, these data were used directly for estimating and projecting maternal mortality ratios. For other Member States a multilevel regression model was developed using available national level data from surveys, censuses, surveillance systems and death registration. This regression model included national income per capita, the general fertility rate and the presence of a skilled attendant at birth (as a proportion of total births) as covariates to predict trends in maternal mortality. Note that numbers of maternal deaths were adjusted upwards by a country-specific fraction, or by 50%, for countries with useable death registration data but without country-specific data on misclassification of maternal deaths, to correct for under-identification of maternal deaths. Note also that the maternal mortality estimates include those HIV deaths occurring in pregnant women or within 42 days of end of pregnancy which were considered to be indirect maternal deaths rather than incidental [11].

### Study variables and source of information

The sources information is WHO [12], UNICEF [13], UNDP [14] and World Bank [15] databases latest available data from 2008 to 2016. Available indicators were sorted and compiled based on developing countries list. The outcome variable was MMR (Maternal mortality ratio) per 100,000 live births; while the determinants were health and socio-economic indicators used by WHO and other organizations.

Determinant factors were classified in to three categories. Socio-economic indicators: GNI, women unemployment rate, public expenditure for health, literacy rate, and age of first marriage [16]. Health care system indicators: Antenatal care coverage, Skilled delivery service coverage, unmet need for contraceptive, contraceptive prevalence rate, total fertility rate, crude birth rate, physician to public proportion, nurse and midwifes proportion rate, safe water supply coverage and improved sanitation.; Disease burden indicators: Tuberculosis incidence, HIV incidence and anemia during pregnancy were used [17]. The variables were selected based on the existing evidence from previous studies.

### Operational definition

According to definitions of World Bank and World Health Organization the following definitions of terms are adopted [10, 12–14, 18].

**MMR:** is the number of deaths among women from any cause related to or aggravated by pregnancy or its management (excluding accidental or incidental causes) during pregnancy, childbirth, or within 42 days of termination of pregnancy, irrespective of the duration or site of the pregnancy, for every 100, 000 live births in a given year or period of time.

**Developing country:** countries with GNI per capital of 4125 USD or less in 2014.

**Antenatal care coverage**: Percentage of women aged 15–49 years that were attended at least once during pregnancy by skilled health personnel (doctor, nurse, or midwife).

**Births attended by skilled health personnel:** Percentage of births that received care from qualified medical personnel.

**Access to an improved water source:** The percentage of population using an improved drinking water source.

**Access to improved sanitation:** Percentage of the population with access to facilities that hygienically separate human excreta from human, animal and insect contact.

**Contraceptive prevalence**: The percentage of women aged 15–49 years, married or in-union, who are currently using, or whose sexual partner is using, at least one method of contraception, regardless of the method used.

**Total fertility rate:** The average number of children a hypothetical cohort of women would have at the end of their reproductive period if they were subject during their whole lives to the fertility rates of a given period and if they were not subject to mortality.

**Density of physicians (per 1000 population):** Number of medical doctors (physicians), including generalist and specialist medical practitioners, per 1000 population.

**Crude birth rate:** The crude birth rate is the annual number of live births per 1000 populations.

**GNI per capita**: Gross National Income (in current US Dollars) divided by midyear population.

**Health expenditure (% GDP):** Level of total expenditure on health (THE) expressed as a percentage of gross domestic product (GDP).

**Adult literacy rate:** Percentage of persons aged 15 years and over who can both read and write.

### Statistical analysis

After the data were obtained from different sources, it was compiled with excel, each variable was checked for completeness and consistency. Whenever a series of data obtained, the most recent one was used. Data was cleaned, coded and exported to SPSS version 21 for Windows, and then exploratory data analysis carried out to check the levels of missing values, presence of influential outliers and normality. The normality of the quantitative variables was checked using the Kolmogorov-Smirnov test. Based on the final compiled data, descriptive analysis of both the independent and dependent variables of interest was performed. The results were presented in the form of tables, texts and figure. Finally, a Pearson or Spearman correlation analysis was performed to ascertain the degree of the relation between MMR and the remaining variables. All associations and tests were said to be significant at *p* < 0.05.

## Results

### Description of variables

A total of 82 developing countries were included in this study. Of which 31 are low incomes countries with GNI per capital of 1045 USD or less and 51 are lower middle income countries with GNI per capital of 4125 USD or less. The mean GNI is 1701(SD = 1249) ranges between 260 and 4280 USD.

Mean of ANC (Antenatal care) utilization is 78% (SD = 28.7) ranging between 41% in Ethiopia to 100% coverage in Korea Democratic Republic. Delivery service utilization ranges from 16% in Ethiopia to 100% coverage in 5 countries with a mean of 62.8 (SD = 30). Only one country had a contraceptive prevalence of 80%, while others have lower contraceptive prevalence than the mentioned figure. Five countries attained 100% safe water coverage and another two countries had attained sanitation coverage of 100%. There are 0.5(SD = 0.8) physicians and 1.4(SD = 2.14) nurses per 1000 population respectively. Ukraine had the highest physician to population ratio with 3.5 physicians per 1000 population while Uzbekistan has the highest nurse to population ratio with 11.9 nurses with 1000 population. The incidence of Tuberculosis (TB) is highest in Lesotho (788 per 100, 000 populations) and lower in West Bank and Gaza which is only 1 per 100,000 population, lower than the average of tuberculosis 195.4(SD = 163) per 100, 000. While HIV incidence is highest in Swaziland (2.36) with a mean of 0.134(SD = 0.36).; The reported prevalence of anemia during pregnancy is between 19% and 64% with a mean prevalence of 37.5% (SD = 14) (Table 1).

**Table 1:** Description of maternal mortality ratio and health and health related indicators among developing countries, 2008–16

Indicators	Mean (SD)	Range
Maternal mortality (deaths per 100, 000 live births)	311(268)	7–1360
Socio-economic variables		
GNI per capital (atlas method)	1701(1249)	260–4280
Unemployment rate	8(8.2)	1–39
Health expenditure (% of GDP)	5.76(2.73)	1.5–13.7
Adult Literacy rate (%)	67(21)	32–100
Early marriage (%)	23(19)	7–45
Health care system related variables		
Antenatal care coverage (%)	78(28.7)	41–100
Skilled delivery coverage (%)	62.8(30)	16–100
Contraceptive Prevalence Rate (%)	35.4(23.4)	4–80
Unmet need prevalence (%)	18(11.4)	5–48
Total fertility per a women	3.8(1.4)	1.3–7.6
Crude birth rate per 1000	29.5(9)	11–49
Ratio of Physician to 1000 population	0.5(0.8)	0.1–3.5
Ratio of Nurse and Midwife to 1000 population	1.4(2.14)	0.1–11.9
Improved water coverage (%)	75(22.7)	40–100
Improved sanitation coverage (%)	47.8(29)	7–100
Disease burden related variables		
TB incidence rate per 100, 000 population	195.4(163)	1–788
HIV incidence rate (15–49 yrs)	0.134(0.36)	0.01–2.36
Anemia during pregnancy prevalence (%)	37.5(14)	19–64

### Maternal mortality ratio (MMR)

MMR ranged from 7 deaths per 100,000 live births in Cabo Verde to 1360 deaths per 100, 000 in Sierra Leon with a mean of 311(SD = 268). It is noted that most countries sharing the highest global maternal death are in this list of developing countries (Table 1).

### Correlation between MMR and variables

Among the socio-economic variables, GNI per capital and adult literacy rate were significantly negatively correlated with MMR, while early marriage significantly correlated positively. All Health care system related variables were significantly correlated with MMR. Skilled delivery service coverage has strong negative correlation with the outcome variable, while total fertility rate per a women and crude birth rate has strong positive correlation with MMR. The strength of relationship in disease burden variable is low (Table 2).

**Table 2:** Correlation between maternal mortality ratio and health and health related indicators among developing countries, 2008–16

Indicators	N	r	*p* value
Maternal mortality (deaths per 100, 000 live births)	82	1	
Socio-economic variables			
GNI per capital (atlas method)	78	−0.586	<0.001
Unemployment rate	72	0.035	0.75
Health expenditure (% of GDP)	79	−0.077	0.48
Adult Literacy rate (%)	76	−0.47	<0.001
Early marriage (%)	65	0.64	<0.001
Health care system related variables			
Antenatal care coverage (%)	75	−0.65	<0.001
Skilled delivery coverage (%)	76	−0.78	<0.001
Contraceptive Prevalence Rate (%)	75	−0.424	<0.001
Unmet need prevalence (%)	69	0.33	0.002
Total fertility per a women	82	0.72	<0.001
Crude birth rate per 1000	80	0.73	<0.001
Ratio of Physician to 1000 population	56	−0.506	<0.001
Ratio of Nurse and Midwife to 1000 population	66	−0.45	<0.001
Improved water coverage (%)	79	−0.32	0.003
Improved sanitation coverage (%)	80	−0.678	<0.001
Disease burden related variables			
TB incidence rate per 100, 000 population	82	0.242	0.027
HIV incidence rate (15–49 years)	64	0.19	0.08
Anemia during pregnancy prevalence (%)	80	0.48	<0.001

## Discussion

This study aimed to assess the correlation between socio economic indicators, health indicators, disease burden indicators and maternal mortality ratio among 82 developing countries. Accordingly it is shown that the highest burden of maternal death belongs to these groups of countries. Moreover the rate of MMR decline in these countries was steady [1]. The ratio of maternal mortality ratio is found very high which ranges from 7 to 1360 deaths per 100,000 live births with a mean of 195.4(SD = 163) per 100,000 live births. Maternal mortality ratio is correlated with socio-economic indicators, health indicators and disease burden indicators of a country. Socio-economic indicators have significant correlation with MMR. This indicates the possible influencing factors beyond routinely studied health care system-related variables. Wide variations in maternal mortality levels exist among different income groups, resource expenditure for health and unemployment level which urges countries to design comprehensive intervention which acknowledges the potential effect of the aforementioned variables for reducing maternal mortality. The finding was supported by many other studies [8, 18]. Maternal mortality ratio differences between developed and developing countries witness the effect of income on maternal mortality [12–15].

It is known that teenage pregnancies carry high risk of mortality. In line with other studies this research found that prevalence of early marriage has significant relationship with maternal mortality [18, 19]. This could be due to the fact that at early age females pelvic is not capable to carry a fetus posing higher risk for obstructed labor and thus, its consequences may be maternal death in the worst scenario. Similarly literacy level was found to be correlated with MMR, which could be due to a multiple effect of knowledge for healthy practice and service utilization. Therefore Provision of information and health promotion about reproductive health services is important for reducing maternal mortality [1, 2].

Health care system associated factors are mostly known determinants of MMR. In line to a study conducted in rural Pakistan staff patterns of health facilities was found to be significantly associated with maternal mortality [20]. The number of physicians and nurses available for every 1000 population has negatively associated with magnitude of MMR. This could be due to the fact that availability of health professional increase availability of maternal care service and improve the quality of existing service and further reduce maternal mortality. Similarly utilization of antenatal care and skilled delivery service has significantly and negatively associated with MMR. This finding is also reported from previous studies [19, 20].

Fertility is an important determinant of maternal mortality, with high fertility levels associated with high maternal mortality [1, 12, 18]. In this study it is noted that total fertility rate has positive significant correlation with MMR which could be due to the fact that the risk of getting complication is proportional to the number of pregnancies a woman will have. A number of other factors which also influence total fertility rate; like crude birth rate, unmet need for contraception and contraceptive prevalence rate has shown to influence MMR. Crude birth rate and unmet need have positive correlation with MMR while CPR has negative correlation.

Access to improved sanitation and safe water supply are other factors observed to be related to maternal mortality. Both are inversely correlated with MMR, which is similar to the previous findings [18–20]. This is due to the fact that access to improved sanitation and safe water decrease the risk of communicable disease which has a larger share of maternal mortality. Therefore along with socio-economic and health care associated variables, access to improved sanitation and safe water supply might explain part of this difference of MMR among developing countries.

The other determinants of MMR are classified as the burden of common diseases. Morbidity levels for relevant conditions in a population significantly influenced maternal deaths. Prevalence of anemia during pregnancy has positively and significantly correlated with MMR. As it was reported from previous studies anemia increase the risk of bleeding and infection which poses higher burden of death [19, 21]. On the other hand the incidence of Tuberculosis in the general population has significantly correlated, while unlike other studies the association between MMR and HIV incidence was not significant [18].

However, variables like unemployment rate of mothers and percentage of health expenditure from GNI were not significantly correlated with MMR. The difference ascribed to share of health expenditure was also reported in another study but the case of unemployment level was opposite to the finding of the research which could be due to low variability among the selected countries [18].

### Conclusion and recommendation

Maternal mortality is a major public health issue in the globe specifically in developing countries due to its high magnitude and lower declining pattern. It is associated with multiples of socio-economic factors, health care system factors, disease burden and their complex interactions. Therefore policy and programmes targeted to improve maternal health and reduce maternal deaths should consider population dynamics, socio-economic influence and health system factors that pose a major risk on mothers.

## Abbreviations

ANC: Antenatal care
CPR: Contraceptive prevalence rate
MMR: Maternal mortality ratio
SPSS: Statistical package for social sciences
TB: Tuberculosis
UNDP: United Nations Development Program
UNICEF: United Nations Children’s Fund
WHO: World Health Organization
